# Supplementary material for: Conditional chemoconnectomics (cCCTomics) as a strategy for efficient and conditional targeting of chemical transmission
Source: eLife. 2024 Apr 30;12:RP91927. doi: 10.7554/eLife.91927 (PMC11060718; doi:10.7554/eLife.91927)
Supplement: Figure 3—source data 3. [file elife-91927-fig3-data3.zip › Figure 3 source data3 part 1.zip!/Figure 3-Source data 3-part1/1063-13-CRISPResso_Report_8cz_fsdr.zip!/CRISPResso_on_1063_15/4b.Insertion_deletion_substitution_locations.pdf]

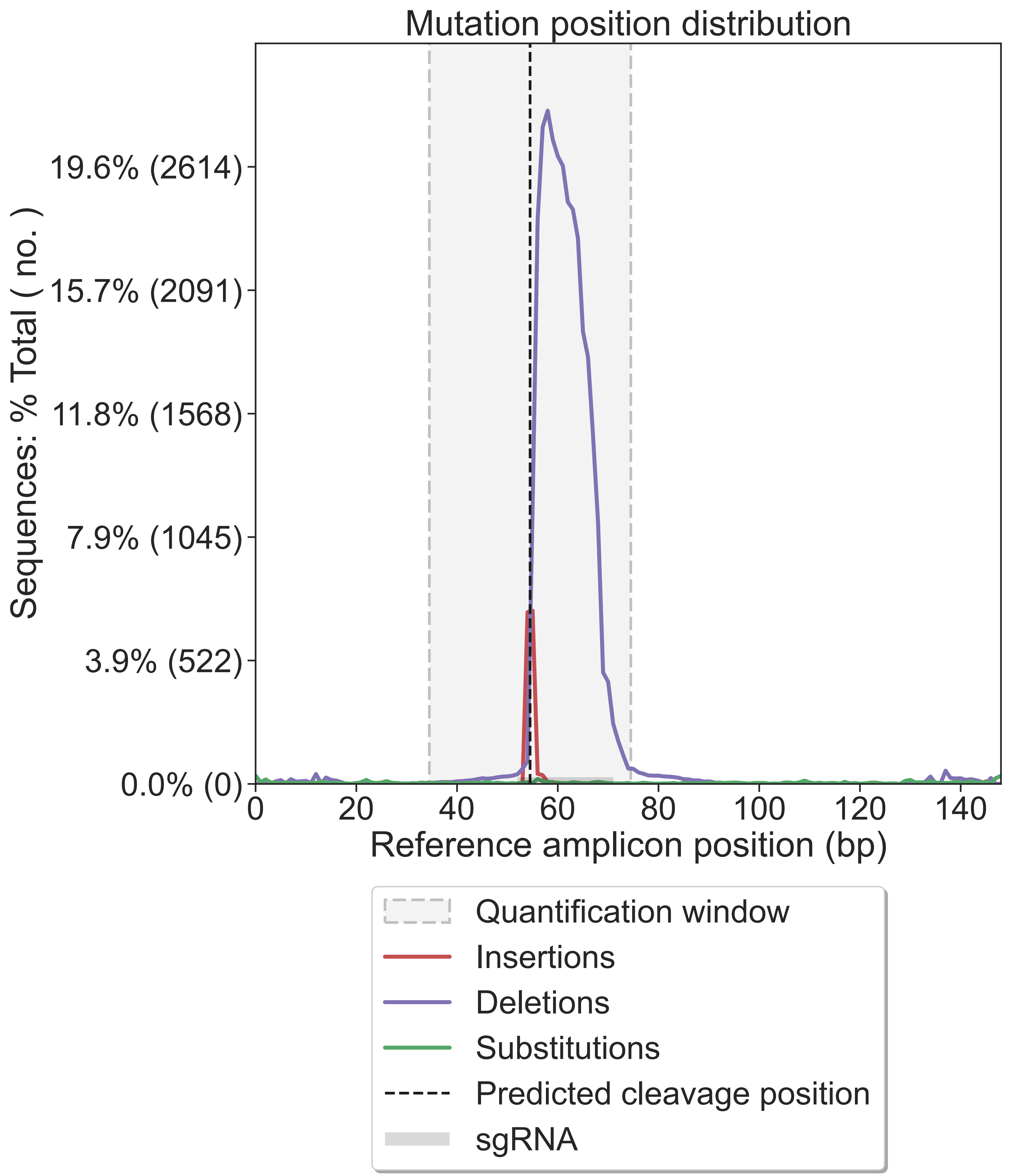
Mutation position distribution
Sequences: % Total (no.)
19.6% (2614)
15.7% (2091)
11.8% (1568)
7.9% (1045)
3.9% (522)
0.0% (0)
0
20
40
60
80
100
120
140
Reference amplicon position (bp)
Quantification window
Insertions
Deletions
Substitutions
Predicted cleavage position
sgRNA
